# A theoretical model for analysing gender bias in medicine

**DOI:** 10.1186/1475-9276-8-28

**Published:** 2009-08-03

**Authors:** Gunilla Risberg, Eva E Johansson, Katarina Hamberg

**Affiliations:** 1 Department of Public Health and Clinical Medicine, Family Medicine, Umeå University SE-90185 Umeå, Sweden; 2 Centre for Gender Excellence, research programme Challenging Gender, Umeå university, SE 90187 Umeå, Sweden

## Abstract

During the last decades research has reported unmotivated differences in the treatment of women and men in various areas of clinical and academic medicine. There is an ongoing discussion on how to avoid such gender bias. We developed a three-step-theoretical model to understand how gender bias in medicine can occur and be understood. In this paper we present the model and discuss its usefulness in the efforts to avoid gender bias. In the model gender bias is analysed in relation to assumptions concerning difference/sameness and equity/inequity between women and men. Our model illustrates that gender bias in medicine can arise from assuming sameness and/or equity between women and men when there are genuine differences to consider in biology and disease, as well as in life conditions and experiences. However, gender bias can also arise from assuming differences when there are none, when and if dichotomous stereotypes about women and men are understood as valid. This conceptual thinking can be useful for discussing and avoiding gender bias in clinical work, medical education, career opportunities and documents such as research programs and health care policies. Too meet the various forms of gender bias, different facts and measures are needed. Knowledge about biological differences between women and men will not reduce bias caused by gendered stereotypes or by unawareness of health problems and discrimination associated with gender inequity. Such bias reflects unawareness of gendered attitudes and will not change by facts only. We suggest consciousness-rising activities and continuous reflections on gender attitudes among students, teachers, researchers and decision-makers.

## Introduction

The concept of gender has been used in the social and humanistic sciences since the 1960's. It was originally introduced to designate how different societies and cultures interpret biological sex [1]. It refers to the constantly ongoing social construction of what is considered 'feminine' and 'masculine' ('doing gender'); a construction based on power and sociocultural norms about women and men [2-5]. The power asymmetry between women and men has been conceptualized as 'the gender order' [6], a structuring principle in society characterized by separation and hierarchy. Sociocultural norms build on a dichotomous thinking about women and men, suggesting innate differences [7]. The concept of gender, on the other hand, implies the possibility of change and negotiation. We all 'do gender' in all kinds of social interactions [2,4,5,8]. In professional everyday life, physicians, too, are 'doing gender'. For example when they ask female patients more than male patients about their family situation [9], physicians are influenced by, and contribute to maintain, the gendered view that family matters are women's issues. An alternative way of doing gender would be to question this view, by asking male patients about their family situation as often as female patients.

In medical research today, the term gender is often used wrongly as being synonymous with biological sex [10]. Sometimes sex is simply replaced by gender even when experiments on animals are described. However, gender is a wider concept than sex and include more than biological differences between women and men [2,4,11,12]. A gender perspective in medicine implies that life conditions, positions in society and societal expectations about 'femininity' and 'masculinity' are to be considered along with biology in professional encounters and relations as well as when theorizing about women and men. An unawareness of gender aspects among health care professionals can lead to gender bias in medicine. Such bias has been reported in research during the last decades and there is an ongoing discussion on how to prevent and avoid it [13,14].

In clinical medicine studies have shown unjustified differences in the investigation and treatment of male and female patients; measures that are not evidence-based. Most research on this subject has been about coronary heart disease [15,16], but there are studies also about many other conditions, for example kidney disease [17], HIV/AIDS [18], colorectal cancer [19], COPD [20], Parkinson's disease [21] and psoriasis [22]. In most cases the bias reported is negative for women, but there are also reports about gender bias affecting men. For example there are reports that men with depression [23] and migraine [24] are not diagnosed and treated properly. In fact, during the last decade a focus on the relation between masculinity and health-hazards has emerged [5,25] and thereby a discussion about the 'mixed blessing' of male gender [26].

In addition to gender bias in the investigation and treatment of patients gender bias occurs in medical education, professional careers, and in medical research as well. Analyses of medical textbooks, curricula, education material and examination questions have revealed stereotypical gender patterns and even open patriarchal views [27-32]. In academic medicine there are reports about discrimination and harassment based on gender among medical students [33,34], physicians [35,36], medical researchers [37,38] and medical faculty [35,37,39]. The discrimination leads to a waste of many women's potential and thereby to a loss for academic and clinical institutions [39]. In medical research gender bias results in a suboptimal scientific rationality: biomedicine did not produce adequate knowledge about a series of important diseases until a gender perspective was used [12]. Thus, gender bias in medicine occurs at many levels and these levels impact on each other.

As a contribution to the ongoing discussion on how to prevent gender bias, the aim of this article is to describe a three-step theoretical model on how gender bias in medicine can occur and be understood, and to discuss the usefulness of the model in the work to detect and understand gender bias.

## Methods

### Empirical material

In our model we use results from three of our empirical gender studies conducted in Sweden as examples. Below these studies are briefly described.

1. Physician teachers' knowledge and attitudes are important for the addressing of gender issues in medical education. At the Umeå Medical School in Sweden, we surveyed 303 (29% women) physician teachers' attitudes towards gender issues via a questionnaire. They were asked to rate their degree of agreement with five statements about the importance of gender in consultation, in tutoring students, and in contact with colleagues, with staff and in research. Women assessed gender more important in all these professional relationships than men did [40,41]. There were also open-ended questions asking for explanations of the ratings and for examples of situations where gender might be of importance. One finding in our content analysis of these open-ended answers was that the physicians perceived gender as difference or sameness and/or as equity or inequity between women and men [42].

2. In Sweden there are no recommendations to treat irritable bowel syndrome (IBS) differently for women and men patients. Still, in a paper case about IBS in a national exam for 289 Swedish residents there were different suggestions about investigations and treatment depending on whether the patient was described as a woman or a man. For example, more x-rays of the colon were suggested for men, while more tranquilizers and advice about life style were suggested for women [43].

3. In a research assessment study, Swedish physicians, especially female physicians, upgraded the scientific accuracy of a fictive qualitative research abstract if the author was said to be a woman [44]. These results suggest that a growing awareness of the male domination in the academic world alerts women to upgrade the achievement of female researchers.

### Theoretical tools

The results of our three empirical gender bias studies brought our thoughts to the long history of feminist theories on the meaning of gender difference and sameness and how it interacts with gender equity and inequity [45,46]. In the analysis in this paper we use these theories of gender as sameness/difference and/or equity/inequity between women and men as analytical tools.

The concepts of sameness/difference and/or equity/inequity are common in gender theory and research, but there is an ongoing discussion, sometimes rather confusing, about how to define, interpret, and apply them.

On an ontological level, the question about sameness/difference is whether men and women are seen as essentially different or not. A question on the practical level, illustrating the ontological dimensions of sameness and difference, would be: Should women have the same rights as men and have access to all sections of society, for example to become surgeons, because they are human beings just as men are, with individual and divergent skills and interests, or because they represent other values than men and therefore have something to add? This question also illustrates that 'sameness', as used in the context sameness/difference, corresponds to diversity within gender.

On the epistemological level there has been a discussion whether sameness/difference are fruitful analytical tools for gender researchers. Difference has been associated with essentialism, and sameness with the risk of reproducing the male norm since 'same' is easily understood as 'same as a man'. A practical view has been to use the concepts as means to bring about knowledge that can be used for change, not as goals. This is the way we use them in our analysis of gender bias in medicine.

The discussion on sameness/difference has sometimes been confused when the term 'sameness' has been exchanged by 'equality'. The opposite of equality is inequality and not difference. The terms equality/inequality relate to whether or not individuals, irrespective of if they are a man or a women, have the same value and are free to develop their personal abilities without (gendered) limitations [46,47]. It is possible to strive for equality and think difference at the same time. Moreover, gender equality/inequality is sometimes mixed up with or used synonymously with gender equity/inequity. Gender equity/inequity has to do with whether there is unfairness, discrimination and harassment on the basis of gender or not. As we see it, striving for equality between women and men could, but does not necessarily, include, striving for equity between women and men. In contrast, aiming at gender equity includes working for gender equality. That is why we use the concepts equity/inequity and not equality/inequality.

In our analysis in this paper we were also inspired by the work by Ruiz and Verbrugge in 1997 [15], where assumed equality or assumed differences between women and men are discussed as a two way view of gender bias in medicine.

### The theoretical model

In our model we develop the theories of gender as sameness/difference and/or equity/inequity in three steps as a way to understand gender bias in medicine. We illustrate our analysis by providing typical examples from the studies above. All quotations in step 1 and step 2 in the model are comments from physician teachers in study 1. Each quotation is identified by gender, age and specialty group of the informant. The specific specialty is not presented for anonymity reasons. (The surgical specialty group includes anesthesiology and intensive care, general surgery, pediatric surgery, hand surgery, neurosurgery, orthopedics, plastic and reconstructive surgery, thoracic surgery, urology, obstetrics and gynecology, gynecological oncology, ophthalmology and otorhinolaryngology. The non-surgical specialty group includes pediatrics, dermatology – venereology, general internal medicine, endocrinology, infectious diseases, respiratory medicine, nephrology, rheumatology, geriatrics, occupational & environmental medicine, clinical physiology, transfusion medicine, clinical neurophysiology, neurology, psychiatry, child & adolescent psychiatry, community and social medicine, diagnostic radiology, oncology, rehabilitation medicine and clinical genetics.)

**Figure 1:**
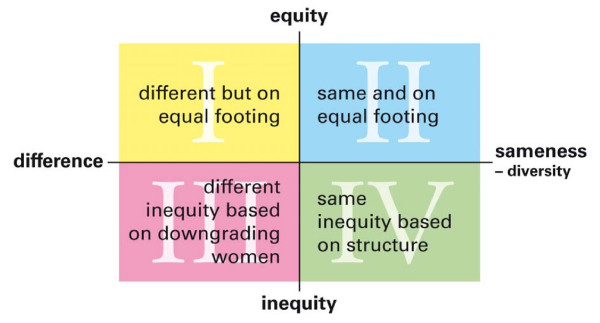
**Assumptions about women and men in relation to perceptions of difference/sameness and equity/inequity**.

**Figure 2:**
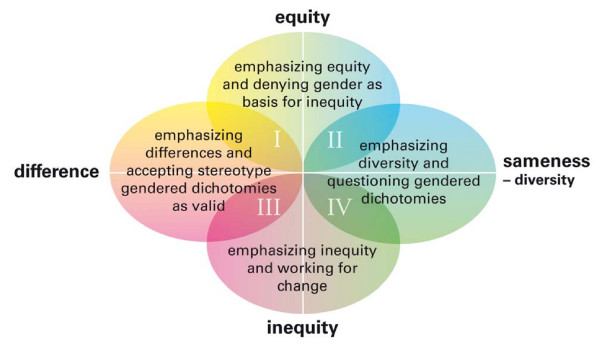
**Approaches to gender in relation to assumptions of difference/sameness and equity/inequity between women and men**.

**Figure 3:**
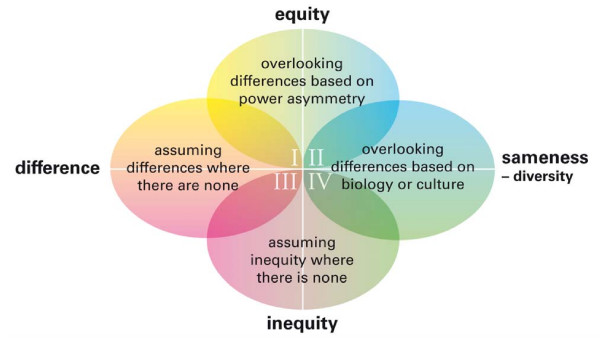
**The risk of gender bias in relation to assumptions about difference/sameness and equity/inequity between women and men**.

Two crossing axes are the basis of the model (as can be seen in figures 1, 2 and 3). The horizontal axis represents the distinction of difference/sameness. 'Difference' stands for the view that there is an inherent difference between the group of women and the group of men while there is sameness within the groups. 'Sameness' indicates the opposite view: that there is a wide diversity within the groups of women and men respectively, and in that sense sameness between them. The vertical axis represents the opposing views on whether there is equity between men and women or not, i.e. whether there is awareness or unawareness of the gender order.

#### Step 1. Assumptions about women and men

In the theoretical model, four fields (I-IV) arise where divergent assumptions about women and men can be identified (figure 1).

I. Women and men are on equal footing but different: *"Men and women doctors can function equally well at our ward. Still, for me, it is easier to talk to male colleagues because we share views on how to investigate and treat patients in most cases. There are fewer irrational elements than in contact with female colleagues." *(male, 59, non-surgical specialty)

II. Women and men are on equal footing and the same: *"I like to think that we are all equal human beings and can understand each other." *(male, 50, non-surgical specialty)

III. Women and men are different and there is inequity emanating from downgrading of women, women's duties and 'female' characteristics:*"It is a shame that women, who are more sensitive and suffer more from pain, are often paid no attention to, and even humiliated, during consultations for pain." *(female, 43, non-surgical specialty)

IV. Women and men are much the same but there is inequity between them, emanating from differences in position, leading to gendered experiences and life conditions:*"I think I would be questioned more in my work by the patients if I were a woman. They never take me for a nurse" *(male, 44, surgical specialty)

#### Step 2. Approaches to the subject of gender

The divergent assumptions about women and men described in step 1 can lead to different approaches to the subject of gender (figure 2).

I and II. Emphasizing equity and denying that gender is a basis for inequities: *"At my workplace all patients are treated in the best way possible and regardless of gender. And, competence, not gender, is what counts at our department." *(female, 59, non-surgical specialty)

II and IV. Emphasising diversity among women and among men and questioning gendered dichotomies: *"In research about differences between women and men there is a tendency to generalize from mean values and forget the distribution within the groups. Are men and women really that different?" *(male, 46, surgical specialty)

III and IV. Emphasizing inequity and working for change: *"Female students are sometimes neglected and often underestimate themselves. This indicates that when tutoring you have to instruct female students 'how to fly' and male students 'how to land."' *(female, 41, unknown specialty)

I and III. Emphasizing differences and accepting stereotype dichotomies about women and men as realities: *"Men and women can never really understand each other, they express themselves altogether differently." *(male, 47, family physician)

#### Step 3. Gender bias

The approaches to the subject of gender described in step 2 all run the risk of being manifested as gender bias (figure 3).

-In fields I and II equity between men and women is presumed. There is a blindness to gender differences in position, influence, life conditions and experiences, which are factors of importance for health and illness and also for the opportunity to make a career. This can lead to upgrading of men and downgrading of women. Perceiving that men and women are treated the same and have equal opportunities, as many teachers did in the open-ended answers [42], opens up for this form of bias. There is an unawareness of discrimination based on gender. The discrimination is seen as individual variations or individual shortcomings.

-In fields II and IV sameness between and diversity within each gender is emphasized. Differences are apprehended as originating in power asymmetry and/or norms. The bias near at hand is to overlook differences connected with biology and disease, for example, differences between men and women in their reaction to drugs, in standard values for blood tests, or in symptoms of coronary disease [48]. This inattention can lead to incorrect treatment of either sex. However, for a long time most research in medicine was performed with male subjects only. Women's biological conditions were overlooked when bodily processes and development of illness were regarded and when symptoms and treatment of disease were recommended [49]. The body of medical knowledge is larger for men than for women. Thus this kind of bias is more likely to affect women.

-In fields III and IV differences in position and power between men and women are acknowledged. In order to obtain equity, there is a risk of upgrading women in an inappropriate way. The women physicians who upgraded female authors in the abstract assessment study [44] demonstrated this kind of gender bias.

-In fields I and III there is an unawareness of the influence of gendered norms and doing-gender processes. There is a risk that differences are assumed where there are none, due to un-reflected dichotomous thinking about women and men. The reasoning of the residents in the paper case study about IBS leading to a different outcome for male and female cases (more x-rays of the colon for men, more tranquilizers and advice about life style for women) [43], exemplifies this type of bias. So does the results of the studies mentioned in the introduction where unmotivated differences in the treatment and investigation of male and female patients are shown [15-24].

Research focusing on biological differences between women and men has produced useful research during the last decades very much on biological differences and has produced a lot of useful research on the subject. However, as illustrated in fields I and III, there is always the risk that small and insignificant differences are exaggerated or that differences are understood strictly in biological terms. Moreover, once knowledge of gender differences in a condition has been established, this might in fact cause gender-biased assessments of individual patients in clinical practice. The differences seen in research between men and women on the group level are often smaller than the differences between individual men in the male group and individual women in the female group. If that fact is disregarded stereotyped generalizations are quickly made. We have labelled this mechanism 'knowledge-mediated gender bias' [43].

## Discussion

This model based on assumptions of difference/sameness and equity/inequity between women and men can be useful in understanding the processes leading to gender bias in medicine. Gender bias can be comprehended as originating in unawareness of gendered norms and doing-gender processes and/or of differences between women and men regarding positions in society, life conditions, life experiences and biology. Consequently, gender bias in medicine can arise from assuming sameness but also of equity between women and men when there are genuine differences to consider in biology and disease, as well as in life conditions, experiences and power. However, it can also arise from assuming differences when there are none, when or if dichotomous perceptions about women and men are understood as valid.

As is illustrated in our model no assumptions about women and men and no approaches to the subject of gender are free from the risk of gender bias. There can be a delicate balance between identifying which differences between women and men are valid and need to be considered to avoid injustices, and which assumed differences are stereotypical ideas leading to bias.

Our analysis on gender bias is an extension of the one made by Ruiz and Verbrugge in 1997: "Like a polarised lens gender bias can arise from two views-one assuming equality where there are genuine differences and the other assuming differences where none may exist" [15]. In the second part of this quotation they oppose equality to difference and contribute to the confusion we discussed when presenting our theoretical tools (see methods). We suppose that they mean "assuming sameness/diversity where there are genuine differences", and "assuming equality where there is inequality". Interpreted in this way, their polarised lens opens up for gender bias arising from more than two views, which is in line with our analysis where the axis of sameness/difference and the axes of equity/inequity are conceptually separated in order to enhance the understanding of different types of gender bias.

### Knowledge of biological differences is not enough to prevent gender bias

Nowadays studying the biological differences between women and men is a growing research field (sometimes named gender-specific medicine [48] despite the focus on biology) and important facts from such studies are included in medical curricula. Thus gender bias originating from the lack of knowledge of biological differences will be reduced. However, the risk of 'knowledge-mediated gender bias' referred to above, will increase; this is a risk physicians have to be aware of.

Moreover, knowledge of biological differences will not prevent the risk of gender bias caused by gendered preconceptions and stereotypes and by unawareness of health problems and discrimination associated with gender inequity. These forms of biases still need to be highlighted in medical research and medical education. We therefore suggest that heads of departments and clinics strongly support gender research focusing the impact of gendered expectations and hierarchies on health problems and diseases, and on physicians' professional role and practice, for example the opportunity for women to advance in academic medicine.

However, these types of bias are associated with attitudes about gender and will not change by facts only. Quite often there is an unawareness of gender attitudes and a belief in objectivity and neutrality among physicians [50]. One of the physician teachers in our questionnaire study [42] expressed it like this: *"I am solely a professional, neutral and genderless." *(male, 40, surgical specialty) This calls for consciousness-rising activities like role-plays and case discussions from a gender perspective in educational programs for students as well as for medical faculty, physicians and medical decision-makers and department chairs. Continuous reflections on gender attitudes should be encouraged in such programs.

### A useful model

The conceptual thinking about gender bias derived from the model can provide ideas and instruments to use for physicians, teachers and researchers in order to handle unawareness of and resistance to gender issues in medicine and to prevent gender bias. We will give some examples:

In clinical situations it is important to evaluate if the investigation and treatment would have been different had the patient been of the opposite sex. If so, is the difference evidence-based or an expression of gender bias? It is also advisable to address gender questions now and then at sittings and rounds at hospital wards and to use the model to perform local follow-ups on investigations and outcome of different diseases and disorders in relation to gender.

In medical education gender issues should be introduced at an early time at medical school and also be part of the educational programs within each specialty. The students get a better understanding of sex and gender and gender bias by being introduced to the theoretical model. It can be used as a basis and starting-point in discussions about diseases where we know that gender bias has been reported for example in coronary heart disease and in depressions. Specific work-shops and interactive discussions with actor patients can be used to address gender attitudes.

As has been discussed by Bickel et al many young women entering medicine and most men conclude that gender equity is achieved by now [39]. They are unaware of or deny injustices based on gender and the different opportunities for men and women that still exist. This gender attitude is important to challenge since it helps maintain gender discrimination by understanding it as something else, for example as individual choices or shortcomings. To introduce the model that we have described in educational programs for students and among faculty and department chairs could be of help. It gives an increased theoretical understanding that differences in career opportunities represent gender bias, not shortcomings of individual women. Such an insight might be an eye-opener for decision-makers, lessen the waste of many women's' potential and strengthen women who otherwise tend to quietly leave or become invisible.

The conceptual ideas of the model can also be helpful when assessing and evaluating a variety of documents such as research programs, research applications or health care and health promotion policies. If researchers, members of research councils and decision-makers get acquainted with the model, they would ask questions like: Which (implicit) assumptions about women and men underlie the document and what type of bias can these assumptions bring about? The same type of questions could be asked concerning individuals, including oneself. How does the individual perceive sameness/difference and equity/inequity between women and men? Which type of gender bias does he/she run the risk of because of these assumptions?

The model can also be applied to and used as a tool to analyse and understand other hierarchical systems than gender, for example class and ethnicity.

## Conclusion

Our model illustrates that gender bias in medicine can arise from assuming sameness and/or equity between women and men when there are genuine differences to consider in biology and disease, as well as in life conditions and experiences. Gender bias can also arise from assuming differences when there are none, when and if dichotomous stereotypes about women and men are understood as valid. This conceptual thinking can be useful for discovering and preventing gender bias in clinical work, medical education, career opportunities and documents such as research programs and health care policies. The strength of the model is the combined focus on knowledge and awareness – are men and women the same/different and are we aware of the gender order? The model can be expanded to other categories of diversity and opens up for future research and other consciousness-rising activities.

## Competing interests

The authors declare that they have no competing interests.

## Authors' contributions

All three authors contributed to the conception, design, and analysis of this article. GR drafted the article and KH and EEJ revised the article critically for important intellectual content. All authors read and approved the final manuscript.
